# Identifying patients with EVEN‐plus syndrome using exome sequencing and clinical feature analysis: A case report

**DOI:** 10.1002/mgg3.2039

**Published:** 2022-09-02

**Authors:** Hua‐Wei Li, Bing‐Xiang Ma, Ya‐Min Kong, Hong Zheng, Xue‐Yuan Zhang

**Affiliations:** ^1^ Department of Pediatrics The First Affiliated Hospital of Henan University of Traditional Chinese Medicine Zhengzhou China

**Keywords:** epiphyseal dysplasia, EVEN‐plus syndrome, *HSPA9*, microtia

## Abstract

**Background:**

The EVEN‐plus syndrome (epiphyseal–vertebral–ear–nose dysplasia plus associated findings) is an extremely rare autosomal recessive inherited disease characterised by specific facial features and skeletal dysplasia. It has a prenatal onset due to defects in the *HSPA9* gene. The syndrome has not been reported previously in China.

**Methods:**

This study reported the characteristics, examination results, diagnosis and treatment of a female case aged 3 years and 3 months.

**Results:**

The patient had global developmental delay and specific facial features, including a prominent forehead, a bilateral auricle deformity, a collapsed nose, a high palatine arch, a short neck and other appearance abnormalities. Her hip joint magnetic resonance imaging (MRI) results showed bilateral femoral head epiphyseal dysplasia with a fork‐shaped malformation at the distal end, and her brain MRI showed white matter myelin dysplasia. *HSPA9* compound heterozygous variants c.882_c.883delAG and c.613A>G were identified by exome sequencing.

**Conclusions:**

This finding expands the spectra of EVEN‐plus syndrome phenotype and pathogenic variants and suggests that c.882_c.883delAG may have a higher distribution frequency in East Asian populations.

## Graphical abstract

This study reports the first case of EVEN‐plus syndrome in China. This child has a typical clinical phenotype with a high palatal arch and the discovery of a ‘trident’ hand, which enriches the phenotypic spectrum of EVEN‐plus syndrome. In addition, the identification of the new mutation p.T205A expands the spectrum of pathogenic variants in the HSPA9 gene.

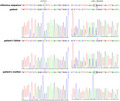

## 1 BACKGROUND

The EVEN‐plus syndrome (epiphyseal–vertebral–ear–nose dysplasia plus associated findings) is an extremely rare autosomal recessive inherited disease characterised by special facial features and skeletal dysplasia; it has a prenatal onset due to defects in the *HSPA9* (MIM: #616854) gene. It has not been reported previously in China (Amiel et al., 1999). The present study reported the case of a female aged 3 years and 3 months with EVEN‐plus syndrome in China. This finding expands the spectra of EVEN‐plus syndrome phenotype and pathogenic variants and suggests that c.882_c.883delAG may have a higher frequency distribution in East Asian populations.

## 2 METHODS

### 2.1 Ethical compliance

This study was conducted in accordance with the Declaration of Helsinki and approved by the ethics committee of the First Affiliated Hospital of Henan University of Traditional Chinese Medicine. Written informed consent was obtained from the parents of the patient.

### 2.2 Case presentation

This case was identified in the First Affiliated Hospital of Henan University of Traditional Chinese Medicine, Zhengzhou City, Henan Province. The child patient (female, aged 3 years and 3 months) was admitted by the outpatient department because she had been ‘found to be motor delayed for about 2 years and 6 months’. The child was admitted to the local hospital with a diagnosis of ‘developmental delay’ at the age of 11 months, when she still could not support herself at four points and could not stand with holding; she was treated intermittently with rehabilitation therapy for about 1 year until she could crawl at the age of 13 months and walk unaided at the age of 20 months.

The child was born at 38 + 4 weeks of age. The mother was G1P1, the delivery was vaginal, the birth weight was 2.67 kg, the fontanelles were closed at 2 years of age and there was a history of asphyxia at birth. The child was hospitalised in a local hospital for 14 days (details unknown) without a history of jaundice. Her mother had vaginal bleeding during pregnancy and had taken oral progesterone. The parents were healthy and not inbred, and there was no family history of hereditary disease. On admission, the child was found to be conscious and mentally well, with a fair response to being called. She was also able to identify simple objects, could not understand and execute simple instructions, could pronounce the ‘a’ sound, could call her mother and father and could count ‘1, 2, 3’. She could use her hands to indicate goodbye and could stand and walk unaided, although she could not run or jump. The child had slightly flexed hips when standing and walking, significant flexion and valgus of both knees, flat and valgus feet and each hand could hold the other. Her flexibility was not good, and pathological signs were not elicited.

**FIGURE 1:**
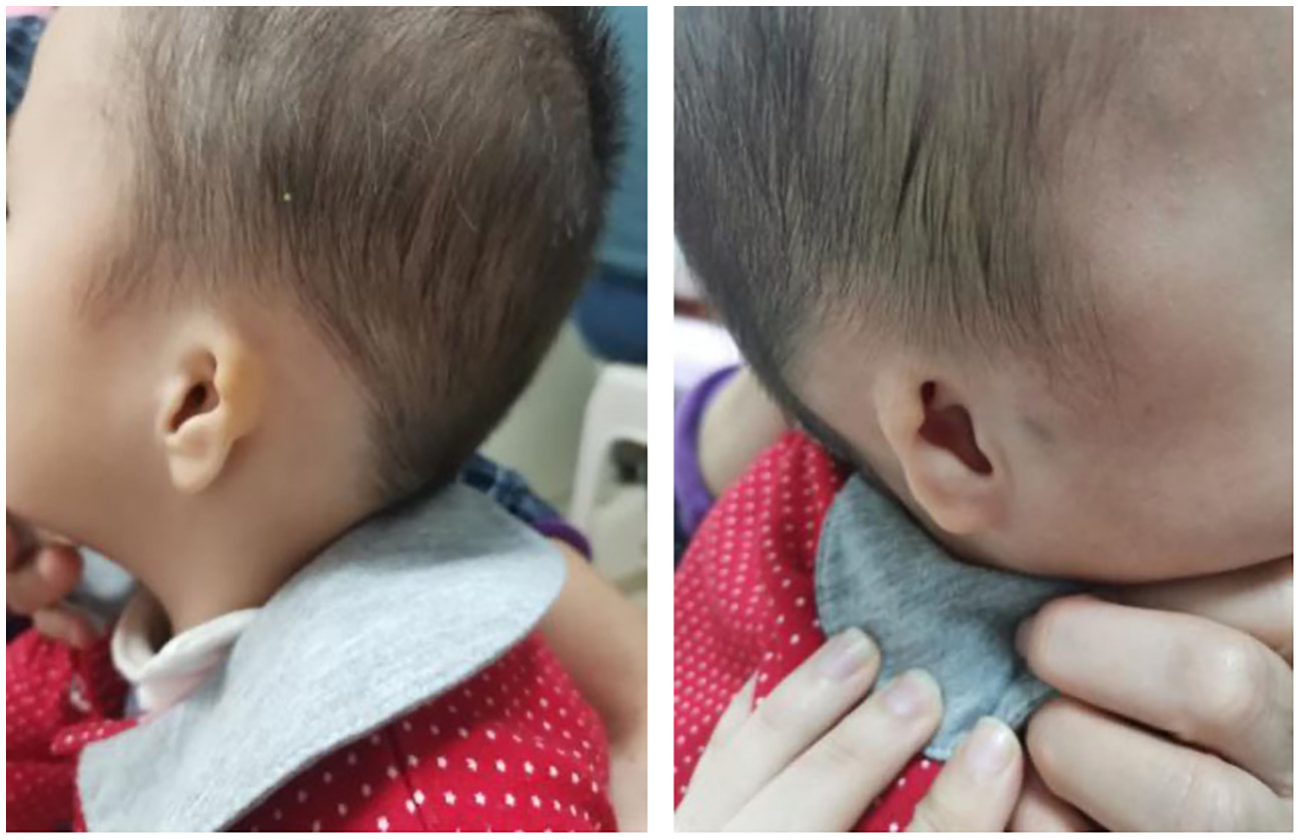
Bilateral auricle deformity of the patient with EVEN‐plus syndrome

The results of a specialist examination were as follows: The patient was 85 cm tall, with a head circumference of 46 cm. She had sparse hair, a prominent forehead, auricular microtia deformity (Figure 1), a collapsed nasal bridge, a high palatal arch, a short neck, natural separation of the index and middle fingers of both hands, eversion of the costal margin and significant flexion and valgus of the knee joint.

She exhibited delayed cognitive and speech development: She could point out simple objects, although she could not understand and execute simple instructions. She could pronounce only the ‘a’ sound, call her mother and father and count ‘1, 2, 3’. She could not communicate or play with children of the same age.

In terms of delayed motor development, she could crawl, sit, stand and walk unaided and walk slowly, although she could not run and jump. She could go up and down stairs with assistance, pinch her hands together and use a spoon to eat, although she was inflexible when using chopsticks. Her hips were slightly flexed when standing and walking, she had significant flexion and valgus of both knees and had flat and valgus feet.

She had abnormal muscle strength and muscle tension: She was uncooperative in a muscle strength examination, she had slightly higher lower‐limb muscle tension and no resistance to passive movement of the shoulder and elbow joints. There was slight contracture of the ankle joint (femoral angle = 80°, popliteal angle = 120°, dorsiflexion angle = 80°), with tendon reflex (+ +), ankle clonus (−) and Barthel sign (+).

The Gesell developmental assessment was as follows: adaptive ability DQ:34.7, gross motor DQ:30.1, fine motor DQ:34.6, verbal ability DQ:39.9, social ability DQ:34.8 and general developmental quotient DQ:34.8, indicating severe developmental delay.

The infant–junior‐high‐school student social life ability scale score was 8 points, indicating mild problems. The S‐S verbal ability ratings were 3–2 (<2 years old) for the comprehension stage and 3–1 (<1.5 years old) for the expression stage.

## 3 RESULTS

**FIGURE 2:**
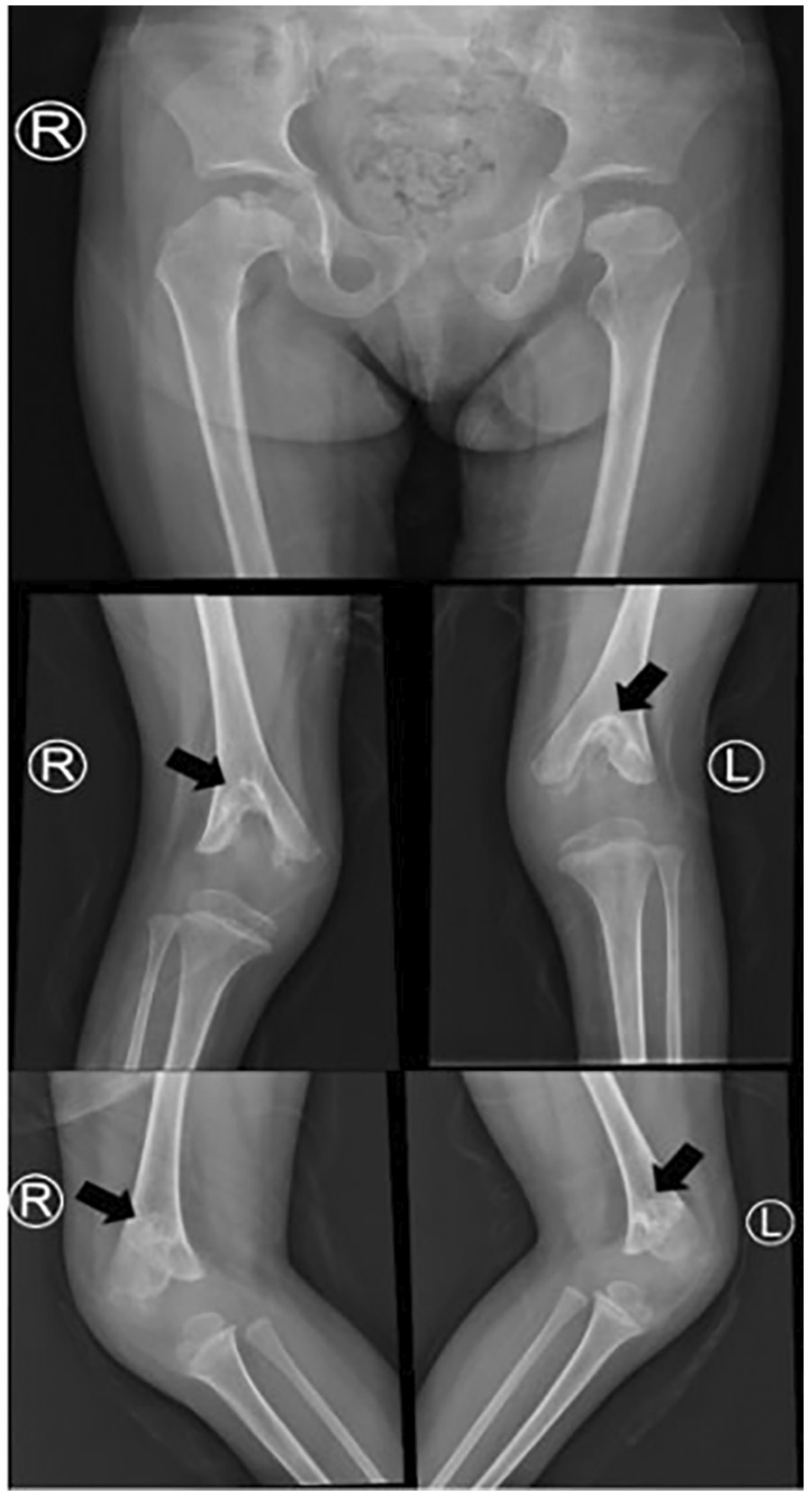
X ray test of lower limb bones in the patient with EVEN‐plus syndrome. The arrows show the ‘fork‐like’ changes in the distal femurs

**FIGURE 3:**
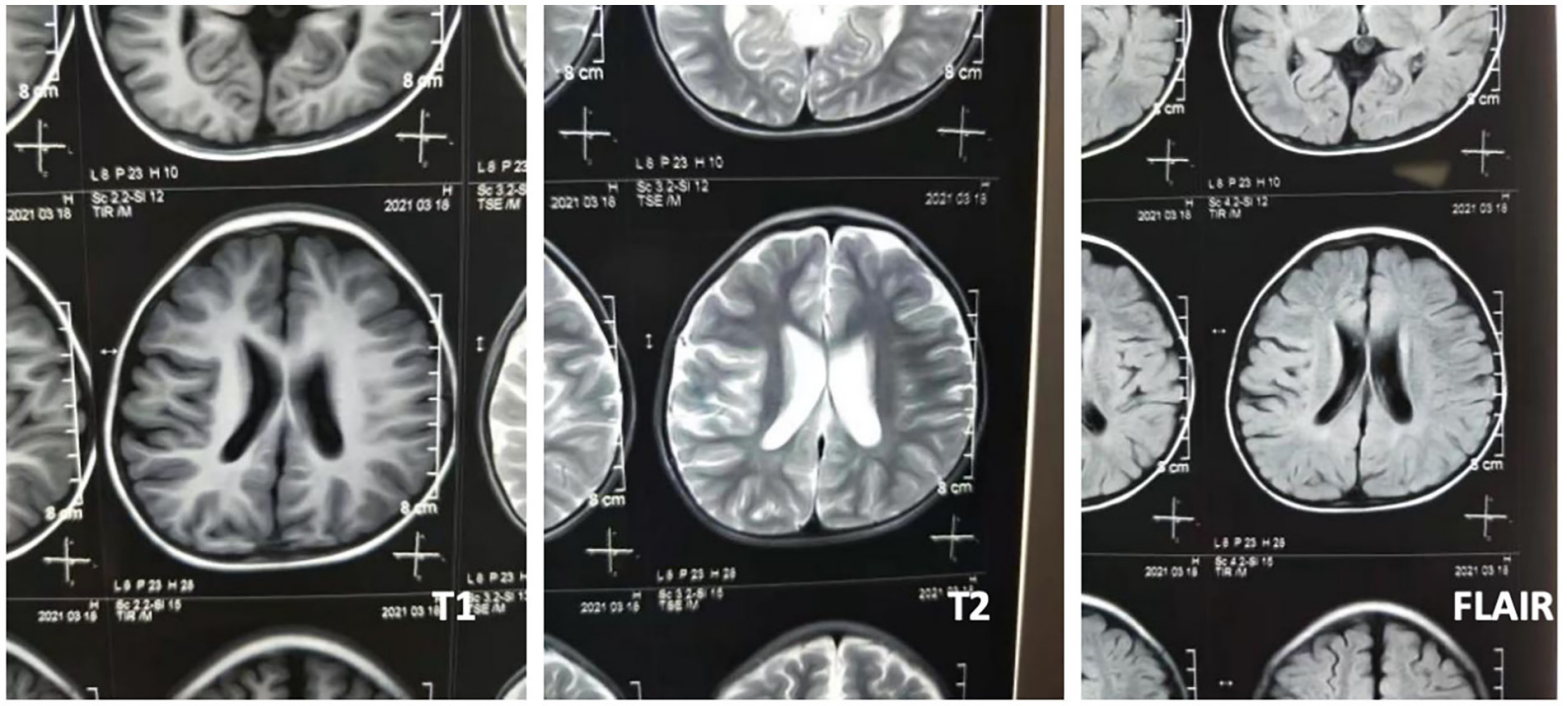
Brain MRI examination in the patient with EVEN‐plus syndrome. Features include: irregular shape of bilateral ventricles, reduction of periventricular white matter, spots of slightly longer T1 and longer T2 signal shadows in bilateral basal ganglia, FLAIR sequence showing high signal, suggesting periventricular white matter softening

The results of the auxiliary examination were as follows: The routine blood biochemical tests and screening of blood amino acid/carnitine profiles and urinary organic acid profiles were not significantly abnormal. The child's electroencephalogram did not show any abnormality, and digital X‐ray imaging suggested symmetrical multiple epiphyseal dysplasia in the hip joint and lower limbs; there was a widening of the joint space and joint cavity effusion and depression of the joint surface of the distal femur, with a characteristic forked shape (Figure 2). The brain MRI results showed irregular morphology of the bilateral lateral ventricles, proximity of the lateral ventricular rim to the cerebral sulcus, reduction of cerebral white matter adjacent to the lateral ventricles, punctate slightly long T1 and slightly long T2 signal shadows in the bilateral basal ganglia region and high signal shadow in the fluid‐attenuated inversion recovery sequence, indicating periventricular leucomalacia lesions or inflammatory sequelae changes (Figure 3).

**FIGURE 4:**
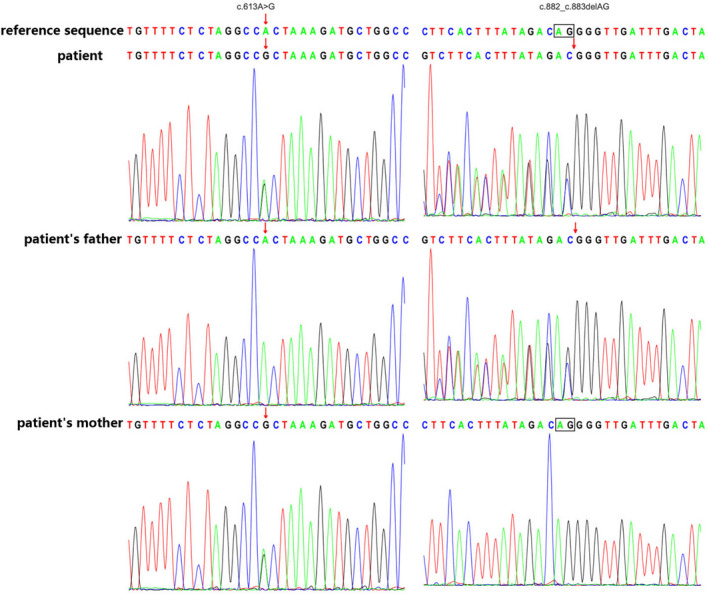
Sanger sequencing in the patient with EVEN‐plus syndrome confirm the *HSPA9* (NM_004134) gene mutations in the patient and her parents

After informed consent was obtained from the child's parents, peripheral blood was collected from both the child and her parents and sent to Beijing Joy Orient Translational Medicine Research Center Co., Ltd. for genomic DNA extraction and exome sequencing. The genetic disease of the variant data was done in a trio model on an online data analysis platform (http://chigene.org). Compound heterozygous variants NM_004134: c.882_c.883delAG, p.T294Tfs*3 (dbSNP: rs772570880) and c.613A > G, p.T205A (dbSNP: rs1304705921) were identified on the *HSPA9* (MIM: #616854) gene of this affected child, and Sanger sequencing was performed to confirm inheritance from the parents respectively (Figure 4).

According to the 2015 guidelines of the American College of Medical Genetics and Genomics, *HSPA9* c.882_c.883delAG and c.613A > G were pathogenic and likely pathogenic variants, respectively, with c.882_c.883delAG being a reported variant identified in patients with EVEN‐plus syndrome (Royer‐Bertrand et al., 2015). *HSPA9* biallelic deficiency was consistent with the EVEN‐plus syndrome (MIM: #616854) phenotype and with the clinical findings of this child. Additionally, we focused on whether the *LONP1* gene was mutated; the result was negative, excluding the possibility that the patient had CODAS (cerebral, ocular, dental, auricular and skeletal anomalies) syndrome.

## 4 DISCUSSION

This study reported the first case of a child with EVEN‐plus syndrome in China. The clinical features of this child were consistent with the core phenotypes of patients summarised by Younger et al. (2020), including specific facial features: synophrys/arched eyebrows, external ear dysplasia, nasal hypoplasia and triangular‐shaped nostrils. The skeletal system abnormalities seen in foetal life are femoral head hypoplasia and a forked distal femur, which are characteristic phenotypes that contribute to the clinical diagnosis. The patient also had a high palatal arch and a ‘trident’ hand, which enriched the phenotypic spectrum of EVEN‐plus syndrome and was useful for the clinical identification of new cases, especially for domestic clinicians. In this case, the child had clinical manifestations of GDD, and her imaging suggested leucomalacia foci. Only one case (1/5) of organic changes in the central nervous system was reported previously (Younger et al., 2020), indicating that central nervous system abnormalities should also be considered during diagnosis.

EVEN‐plus syndrome is attributed to biallelic mutations in *HSPA9*. HSPA9 acts as a mitochondrial chaperone protein (mortalin) and assists in the proper folding of proteins (Kang et al., 1990; Schneider et al., 1994); additionally, it plays a role in cellular senescence, immortalisation/carcinogenesis, neurodegeneration, oxidative stress protection, haematopoiesis and viral replication (Dores‐Silva et al., 2015). The frameshift mutation c.882_883delAG found in this study was earlier identified in a Korean patient reported by Royer‐Bertrand et al. (2015). For the previously reported pathogenic variants p.R126W and p.Y128C, Moseng et al. (2019) found (via in vitro assays) that residues 126 and 128 are located on the surface of the nucleotide‐binding domain (NBD), while p.R126W and p.Y128C are located on the surface of the NBD. These variants reduce the thermostability of the NBD, leading to a reduced affinity for the domain indirect head peptide; thus, they may reduce the activity of ATPase (Moseng et al., 2019).

The unreported variant p.T205A was identified in our patient. It is located in a helix structure with an unknown specific function (UniProtKB: P38646), and the mechanism by which it causes abnormal protein function remains to be confirmed by in‐depth molecular biological basic studies. Among the three patients with EVEN‐plus syndrome reported by Royer‐Bertrand et al. (2015), two cases were found to be homozygous for variant p.R126W, but the central nervous system manifestations seen clinically were significantly different, with only one case with developmental delay and corpus callosum dysplasia. This indicates that there is clinical phenotypic heterogeneity in EVEN‐plus syndrome, that is there is no strict association between pathogenic variant sites and clinical phenotypes, which should be noted in the diagnosis and identification of new cases.

Although EVEN‐plus syndrome is extremely rare, it needs to be differentiated from CODAS syndrome, which is a rare autosomal recessive disorder caused by a defect in the *LONP1* gene; CODAS also has a similar phenotype of developmental delay, overfolded and shrunken external ears, a flattened and hypoplastic nasal bridge and hypophysis of the extremities. Therefore, early genetic testing is recommended for a definitive diagnosis and a differential diagnosis. In addition, since the abnormal skeletal development of EVEN‐plus syndrome is seen in the foetal stage, for families with such findings in prenatal screening, it is recommended that both parents undergo genetic screening for *HSPA9* and *LONP1*; this is of positive significance for eugenics and subsequent genetic counselling.

## 5 CONCLUSION

This study reported the first case of EVEN‐plus syndrome in China. The child had a typical clinical phenotype with a high palatal arch, and the discovery of a ‘trident’ hand enriches the phenotypic spectrum of EVEN‐plus syndrome. In addition, the identification of the new mutation p.T205A expands the spectrum of pathogenic variants in the *HSPA9* gene.

## AUTHOR CONTRIBUTIONS

Conception and design: Hua‐Wei Li. Administrative support: Bing‐Xiang Ma. Provision of study materials or patients: Ya‐Min Kong. Collection and assembly of data: Hong Zheng. Data analysis and interpretation: Xue‐Yuan Zhang. Manuscript writing: All authors. Final approval of manuscript: All authors.

## FUNDING INFORMATION

This study was funded by the following: National Natural Science Foundation of China (No. 81973904), Henan Province Traditional Chinese Medicine Scientific Research Project (No. 2019JDZX2006), Traditional Chinese Medicine Discipline Construction of Henan Province Key Discipline Construction (No. STG‐ZYXKY‐2020023). Funding agencies did not play a role in study design, data collection, analysis and interpretation, and manuscript writing.

## CONFLICT OF INTEREST

All the authors had no any personal, financial, commercial or academic conflicts of interest separately.

## ETHICS APPROVAL AND CONSENT TO PARTICIPATE

This study was conducted in accordance with the Declaration of Helsinki and approved by the ethics committee of The First Affiliated Hospital of Henan University of Traditional Chinese Medicine. Written informed consent was obtained from the parents of the patient.

## CONSENT FOR PUBLICATION

Written informed consent for publication of identifying images and clinical details was obtained from the parents of the patient.
